# Rapid Assessment of Stony Coral Richness and Condition on Saba Bank, Netherlands Antilles

**DOI:** 10.1371/journal.pone.0010749

**Published:** 2010-05-21

**Authors:** Sheila A. McKenna, Peter Etnoyer

**Affiliations:** 1 Center for Applied Biodiversity Science at Conservation International, Arlington, Virginia, United States of America; 2 Mission Blue, Alameda, California, United States of America; 3 The Ecology Centre and Commonwealth Facility for Applied Environmental Decision Analysis (AEDA), The University of Queensland, Brisbane, Australia; 4 Center for Coastal Environmental Health and Biomolecular Research, National Oceanic and Atmospheric Administration, Charleston, South Carolina, United States of America; Institut Pluridisciplinaire Hubert Curien, France

## Abstract

The benthic habitats of Saba Bank (17°25′N, 63°30′W) are at risk from maritime traffic, especially oil tankers (e.g., anchoring). To mitigate this risk, information is needed on the biodiversity and location of habitats to develop a zone use plan. A rapid survey to document the biodiversity of macro-algae, sponges, corals and fishes was conducted. Here we report on the richness and condition of stony coral species at 18 select sites, and we test for the effects of bottom type, depth, and distance from platform edge. Species richness was visually assessed by roving scuba diver with voucher specimens of each species collected. Coral tissue was examined for bleaching and diseases. Thirty-three coral species were documented. There were no significant differences in coral composition among bottom types or depth classes (ANOSIM, P>0.05). There was a significant difference between sites (ANOSIM, P<0.05) near and far from the platform edge. The number of coral species observed ranged from zero and one in algal dominated habitats to 23 at a reef habitat on the southern edge of the Bank. Five reef sites had stands of *Acropora cervicornis*, a critically endangered species on the IUCN redlist. Bleaching was evident at 82% of the sites assessed with 43 colonies bleached. Only three coral colonies were observed to have disease. Combining our findings with that of other studies, a total of 43 species have been documented from Saba Bank. The coral assemblage on the bank is representative and typical of those found elsewhere in the Caribbean. Although our findings will help develop effective protection, more information is needed on Saba Bank to create a comprehensive zone use plan. Nevertheless, immediate action is warranted to protect the diverse coral reef habitats documented here, especially those containing *A. cervicornis*.

## Introduction

**Figure 1:**
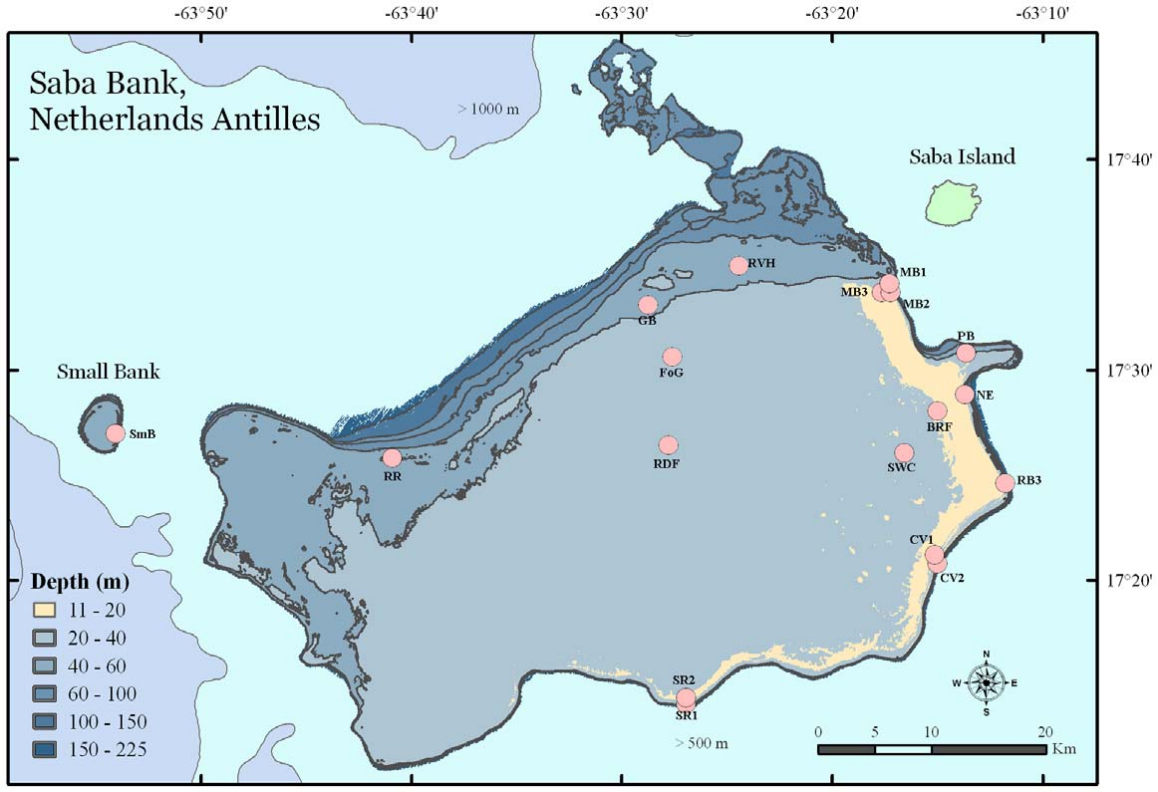
Map of Saba Bank, Netherlands Antilles. Sites assessed during the January 2006 survey are indicated by circles with corresponding name abbreviations (BrF, Brown Fields; CV1, Coral Gardens first location; CV2, Coral Gardens second location; GB, Grouper Bank; MB1, Moonfish Bank first location; MB2, Moonfish Bank second location; MB3, Moonfish Bank third location; NE, North East Reef; PB, Poison Bank; RB3 Redman Bulge; RDF, Red Flats; RR, Rhodolith Reef; RVH, Rendez Vous Hill; SR1, South Reef first location; SR2, South Reef second location; SWC, Seaweed City; and RdF, Red flats. One site was assessed on Small Bank (SmB). The light green polygon depicts the island of Saba. A pale band of color representing 11–20 m depths represents a “fore reef” extending 50 km along the east and southeast perimeter of the Bank.

Saba Bank (17°25′N, 63°30′W; Figure 1) is an elliptically shaped, completely submerged bank located in the windward Netherlands Antilles. The nearest landmass is the small volcanic island of Saba (total area of 13 km^2^), across a deep-water channel 5 km to the northeast. Eighty percent of Saba Bank is encompassed by the Exclusive Economic Zone of the Netherlands, including a small neighboring seamount to the west of Saba Bank called Small Bank. The other 20% of Saba Bank lies within the territorial waters of Saba Island. Saba Bank is the largest atoll in the Atlantic Ocean and one of the three largest atolls on earth, measuring 65 by 40 km in length and width [1]. The area within the 200 m isobath is 2200 km^2^. A large portion of the Bank, approximately 225 km^2^ in area, has a depth range of seven to 20 m and contains extensive coral reefs [2]. The middle of Saba Bank is characterized by hard substrate or “pavement” covered by a veneer of sand that is colonized by algae, gorgonians, and sponges. This area lacks the structural complexity and rugosity found on the reef areas of the Bank [2], [3].

Numerous stony coral species (Orders Scleractinia, Capitata and Filifera) have been recorded from Saba Bank. The most extensive survey was conducted by Van der Land in 1977 where 25 species were documented from 17 reef and plateau (also called “lagoon”) stations [2]. Two subsequent studies [1], [4] were less extensive with fewer sites sampled and no specimens collected for archival purposes. A total of 27 species were recorded in seven reconnaissance dives on the northeast portion of the Bank in 1996 [1]. Sixteen stony coral species were documented during a post hurricane rapid assessment of three northeast reef areas of Saba Bank in 2003 (Table 1) [4].

**Table 1:** Stony coral species documented from Saba Bank.

Class	Order	Family	Genus-species-Author-Year	Year of study			
				1977	1996	2003	2006
Anthozoa	Scleractinia	Acroporidae	*Acropora cervicornis* (Lamarck, 1816)	X	X	X	X
		Agarciidae	*Agaricia agaricites* (Linnaeus, 1758)	X	X	X	X
			*Agaricia grahamae* Wells, 1973				X
			*Agaricia humilis* Verrill, 1902				X
			*Agaricia lamarcki* (Edwards &Haime, 1851)		X		X
			*Agaricia* sp.				X
			*Helioseris cucullata* (Ellis & Solander, 1786)		X		X
		Astrocoeniidae	*Stephocoenia intersepta* (Lamark, 1816)	X	X	X	X
		Caryophyllidae	*Eusmilia fastigiata* (Pallas, 1766)	X	X		X
		Dendrophylliidae	*Tubastraea coccinea* Lesson, 1829				X
		Faviidae	*Colpopyllia natans* (Houttuyn, 1772)	X	X	X	X
			*Diploria clivosa* (Ellis &Solander, 1786)	X			
			*Diploria labyrinthiformis* (Linnaeus, 1758)	X	X	X	X
			*Diploria strigosa* (Dana, 1846)	X	X	X	X
			*Favia fragum* (Esper, 1795)				X
			*Manicina areolata* (Linnaeus, 1758)	X			X
			*Montastraea annularis*(Ellis & Solander, 1786)	X	X		X
			*Montastraea cavernosa* (Linnaeus, 1786)	X	X	X	X
			*Montastraea faveolata*(Ellis & Solander, 1786)		X	X	X
			*Montastraea franksi* (Gregory, 1895)		X	X	X
			*Montastraea* sp.				X
			*Solenstrea bournoni* Milne Edwards & Haime, 1848	X			
			*Solenstrea* sp.				X
		Meandrinidae	*Dendrogyra cylindricus* Ehrenberg, 1834	X	X		X
			*Dichocoenia stokesi* Milne Edwards & Haime, 1848	X	X	X	X
			*Meandrina brasiliensis* (Milne Edwards & Haime 1848)				X
			*Meandrina meandrites* (Linnaeus, 1758)		X		X
		Mussidae	*Isophyllastrea rigida* (Dana, 1846)	X	X		X
			*Isophyllia sinuosa* (Ellis & Solander, 1786)	X	X		X
			*Mussa angulosa* (Pallas, 1766)	X	X		X
			*Mycetophillia danaana* Milne Edwards & Haime 1849		X		
			*Mycetophillia lamarckana* Milne Edwards & Haime 1848	X			
			*Mycetophyllia* sp.				X
			*Scolymia lacera* (Pallas, 1766)	X			
			*Scolymia* sp.				X
		Pocilloporidae	*Madracis asperula* (Milne Edwards & Haime, 1849)	X			
			*Madracis auretenra* (Locke, Weil & Coates, 2007)				X
			*Madracis decactis*(Lyman, 1859)		X	X	X
			*Madracis mirabilis* (sensu Wells, 1973)		X		
			*Madracis* sp.				X
		Poritidae	*Porites astreoides* (Lamarck, 1816)	X	X	X	X
			*Porites divaricata* (Lamarck, 1816)		X		X
			*Porites* sp				X
		Siderastreidae	*Siderastrea radians* (Pallas, 1766)				
			*Siderastrea sidera* (Ellis & Solander, 1786)				
			*Siderastrea* sp.		X	X	X
Hydrozoa	Capitata	Milleporidae	*Millepora alcicornis* Linnaeus, 1758	X	X		X
			*Millepora complanata* Lamarck, 1816		X	X	
			*Millepora squarrosa* Lamarck, 1816				
			*Millepora* sp.			X	
	Filifera	Stylasteridae	*Stylaster roseus* (Pallas, 1766)				X
			*Stylaster* sp				X
Unknown						X	

Species recorded during this study 2006 and previous ones in 1977, 1996, and 2003 [1], [2], [4].

Although the habitats of Saba Bank are presumed to be free from land based threats (e.g. sedimentation and pollutants from run-off), human impacts are evident from maritime and fishing activity. Maritime traffic is high. Cargo ships, freighters, oil tankers, cruise ships and yachts transit over the bank. Of particular concern is the anchoring activity of oil tankers waiting to enter trans-shipment facilities on St. Eustatius [1]. Anchor damage has been reported on the bank [5] and the chains are known to cause extensive physical damage to coral reefs [6], [7]. Other potential damages from maritime traffic include oil spills and dispersants, ship grounding, and collisions.

Fishers from Saba Island and neighboring Caribbean islands target snapper and lobsters using fish pots that can damage coral habitat [1]. Other threats to Saba Bank include hurricanes and bleaching events [4], [8]. Bleaching and disease were postulated as the causes of a documented decline in live coral cover of reefs on the eastern edge of the bank [4], [9]. A severe bleaching event occurred in 2005 prior to our survey (January 2006). Bleaching of corals was first noted in the Dutch Windward Islands (Saba, St. Maarten and St. Eustatius) in late August 2005 and continuing to mid-November 2005 [8].

To mitigate the risks to corals from the maritime activity on Saba Bank, information is needed on the biodiversity and location of sensitive habitats to develop a zoning plan for their protection. A rapid survey to document the species richness of macro-algae, sponges, corals, and fishes was conducted in January 2006 by a multi-disciplinary team. Here we report on the stony coral species from 18 sites. Coral condition, bleaching, disease and evidence of physical disturbance were also examined. This survey was preliminary, conducted one year before high resolution mapping activities were carried out by the Royal Dutch Hydrographic Survey using multi-beam echo sounder technology.

Species richness and condition of stony corals were assessed by a pair of roving scuba divers. Site selection was non-random, intended to cover a broad range of localities with special attention to known or hypothesized reef areas. Sites were categorized by dominant bottom type (pavement or reef), by depth class (deep ≥30 m, mid-depth 25–29 m, and shallow <24 m) and by distance from platform edge (greater to or less than 500 m). Multivariate analyses were employed using non-metric multi-dimensional scaling (MDS) and hierarchical clustering techniques on an underlying Bray-Curtis dissimilarity matrix of presence/absence data. Analysis of Similarities (ANOSIM) Global R statistic was used to test for differences among the *a priori* group designations (i.e. bottom type, distance from the platform edge, and depth class). Similarity profile (SIMPROF) *Pi* statistics were used to test for the null hypothesis of “no structure” in the data when compared to other Caribbean localities [10].

## Results

### Coral Species Richness

**Figure 2:**
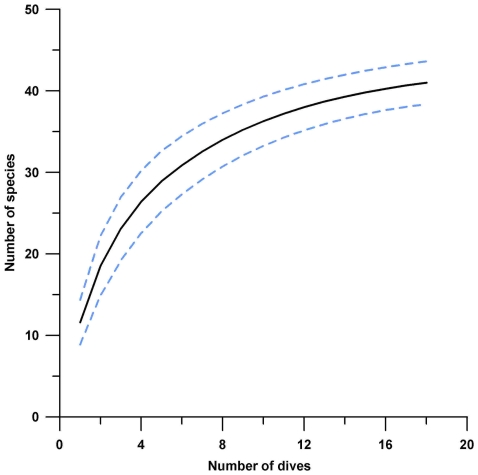
Expected species accumulation curve for stony corals on Saba Bank. The graph shows Mao Tau (S_obs_) values (black solid line) with 95% confidence intervals (dashed light blue lines) based on incidence data from 18 dives. The curve appears to be approaching asymptote, indicating the most common species were collected. More species of coral are likely to be found, through more exhaustive sampling effort and deeper exploration of Saba Bank.

A total of 33 coral species were documented across the 18 sites on Saba Bank (n = 17) and Small Bank (n = 1, Fig. 1). The expected species accumulation curve (Mao observed) appeared to approach asymptote after 18 dives, suggesting most of the common species were collected (Fig. 2). Species richness estimates (Jack1 and Chao1) predict somewhere between 42 and 47 total stony coral species on Saba Bank. More species of coral are likely through more exhaustive sampling effort, particularly in deeper water, and less accessible parts of Saba Bank. Sixty-two coral specimens were collected as vouchers for taxonomic verification or resolution. These were curated at Smithsonian National Museum of Natural History. In a few instances specimens were resolved to genus level only (i.e. *Agaricia* sp., *Mycetophyllia* sp., *Scolymia* sp., *Siderastrea* sp.). Combining our findings with that of other studies [1], [2], [4], a total of 43 species have been documented from Saba Bank (Table 1).

The number of coral species observed ranged from one in the algal dominated habitat of Seaweed City (SWC) to 23 in coral dominated or reef habitat of South Reef first location (SR1). At the algal dominated site Field of Greens (FoG), no coral species were observed so it was excluded from statistical analysis. Five reef sites had stands of *Acropora cervicornis*, a critically endangered IUCN red listed species: Conch Valley second location (CV2) Moonfish Bank first location (MB1) Moonfish Bank third location (MB3), (SR1), and South Reef second location (SR2). A dead stand of *A. cervicornis* was recorded at Conch Valley first location (CV1) with no live colonies observed. One colony of the introduced coral species, *Tubastrea coccinea* from the Indo-Pacific was observed at CV1. *Siderastrea siderea* was the most common species (at 14 of 18 sites) followed by *Montastraea faveolata* (at 13 of 18 sites). Large reef building corals *Montastraea cavernosa*, *M. faveolata*, *Diploria labyrinthiformis* and *D. strigosa* occurred on reef sites. Observed colonies of *Favia fragum, Manicina areolata,* and *Millepora alcicornis* on plateau sites were small in size.

**Figure 3:**
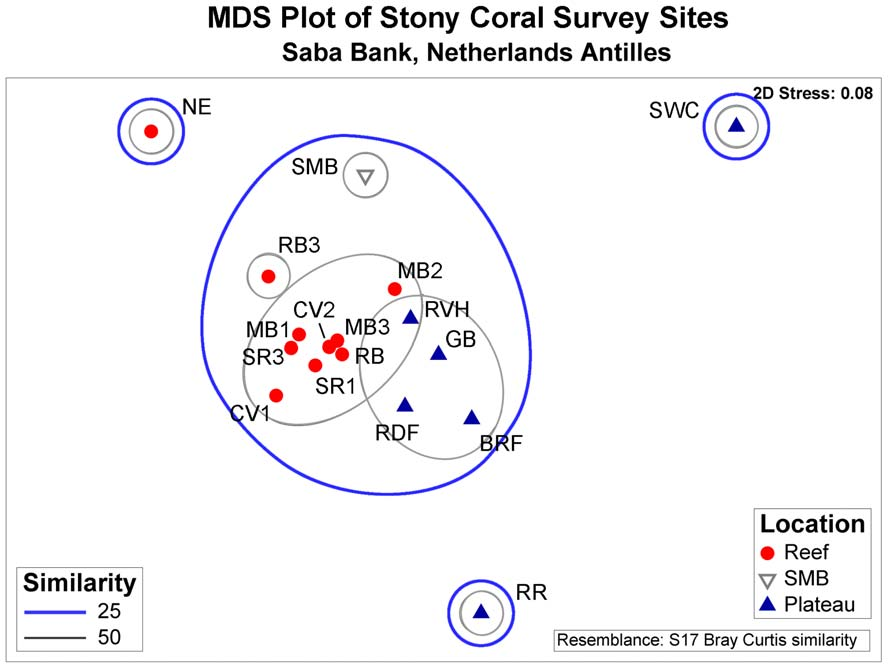
Multi-dimensional scaling plot of similarity in coral species composition at sites sampled during survey. Sites on Saba Bank (n = 16) include the six plateau sites (BrF, GB, RDF, RR, RVH, and SWC) and the ten reef zone sites (CV1, CV2, MB1, MB2, MB3, NE, PB, RB3, SR1, and SR2). One site was sampled on the Small Bank (n = 1), a small neighboring seamount, was not categorized into either class and treated as an independent site from the sites sampled on Saba Bank.

Coral species composition did not differ significantly between bottom types (pavement or reef) (ANOSIM Global R = 0.06, P = 0.18) or among depth classes (ANOSIM Global R = 0.02, P = 0.39), but there was a significant difference in species composition between sites near (<500 m) and far (>500 m) from the platform edge (ANOSIM Global R = 0.497, P = 0.002), Fig. 3). Ten reef sites (NE, RB3, SR1, SR2, MB1, MB2, MB3, PB, CV1, CV2) were significantly different from the six plateau sites (RR, SWC, RDF, GB, BRF, and RVH) (ANOSIM, Pairwise R = 0.494, P = 0.002).

Separation between the groups was along gradients of richness and rarity (Fig. 3). Reef sites clustered to the left, and harbored 15–20 stony coral species. Plateau sites clustered to the right, and harbored 8–11 species. The gradient in rarity was evident from the periphery of the plot to the center. Sites at the periphery had low richness, and rare species. Rare species were defined as species occurring in only one or two sites. Sites at the center of the plot were high richness, with rare species, reef species, and some species shared between reef and plateau sites.

The average Bray-Curtis dissimilarity between reef and plateau sites was high, 72%. *Diploria labryrinthiformis, Eusmilia fastigiata, Meandrina meandrites*, and *M. faveolata* each contributed more than 5% to the dissimilarity between habitats. These four coral species were typical of reef sites, responsible for 40% of the similarity within reef sites. Reef sites were more similar to each other (average 53%) than plateau sites (average 28%). *Agaricia agaricities* was typical of plateau sites, contributing 31% to the average resemblance within plateau sites. *Dichocoenia stokesi, M. cavernosa*,and *S. siderea* each contributed 16%. All the species found on plateau sites also occurred on reef sites, but all observed colonies were apparently smaller (∼10 cm) in plateau sites.

**Figure 4:**
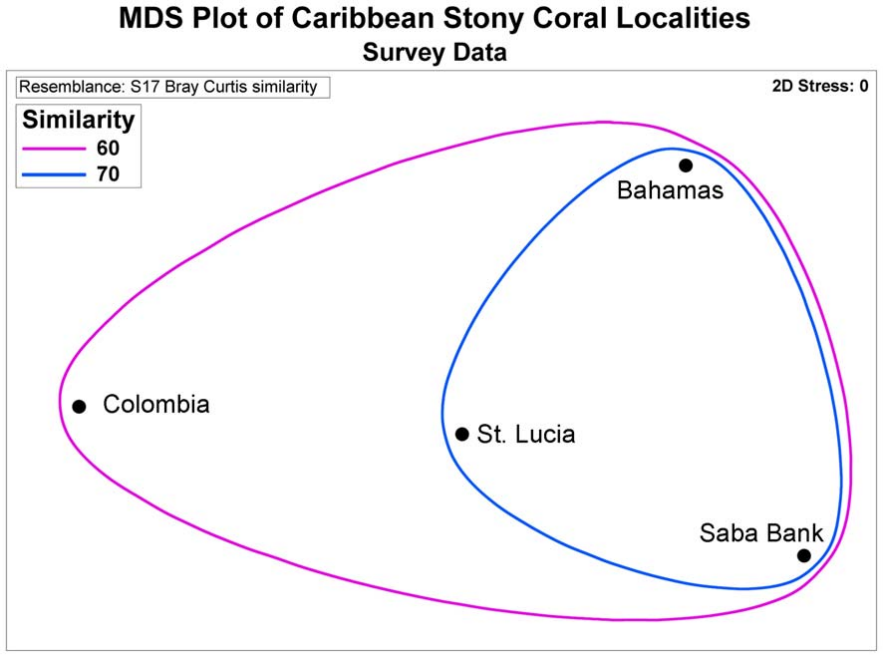
Multi-dimensional scaling plot of similarity in coral species composition between Saba Bank and other Caribbean locations. There was no difference in coral species assemblages among sites sampled on Saba Bank including the one site on Small Bank and the three Caribbean locations (SIMPROF, P>0.05). Sites were 60% similar.

Coral species assemblages on Saba Bank were not significantly different from coral assemblages in Colombia, St. Lucia, and the Bahamas (SIMPROF, P>0.05). Altogether, the four Caribbean coral assemblages were more than 60% similar, with the westernmost site (Colombia) being the most different, and the easternmost sites (St. Lucia and the Bahamas) being most similar (Fig. 4) to Saba Bank.

### Bleaching and disease

Bleaching of coral colonies was noted at 14 out of 17 (or 82%) sites assessed with 43 colonies exhibited discoloration of their tissue (Table 2). All three AGGRA (Atlantic and Gulf Rapid Reef Assessment) categories of coral tissue discoloration (bleached, pale and partially bleached) were recorded. The number of colonies exhibiting bleaching by site ranged from zero at CV2, RR and SWC to seven at GB. The most frequently observed coral species to bleach was *A. agaricites*. There appeared to be a pattern for more colonies to be bleached on sites located in the northern and northeastern sides of Saba Bank. Only three coral colonies exhibited symptoms indicative of coral disease. These included one colony of *D. strigosa* infected with white band disease at SR2 and two colonies of *M. faveolata* with one exhibiting symptoms of white plaque and the other of black band at CV1.

**Table 2:** Species of coral colonies observed by site to exhibit bleaching with severity of tissue discoloration recorded.

Site	# of colonies	# of species	Species	Severity
NE	2	2	*Montastraea faveolata*	Bleached
			*Agaricia agaricites*	Bleached
SMB	1	1	*Agaricia sp*	Bleached
RB3	1	1	*Siderastrea siderea*	Partially Bleached
GB	6	4	*Agaricia agaricites*	Bleached
			*Agaricia agaricites*	Partially Bleached
			*Montastraea faveolata*	Pale
			*Siderastrea siderea*	Partially Bleached
			*Siderastrea siderea*	Bleached
			*Manicina areolata*	Bleached
RVH	5	3	*Agaricia agaricites*	Partially Bleached
			*Agaricia agaricites*	Bleached
			*Isophyllastrea rigida*	Partially Bleached
			*Mycetophyllia sp.*	Pale
			*Mycetophyllia sp.*	Pale
SR1	3	3	*Agaricia agaricites*	Bleached
			*Diploria labyrithiformis*	Pale
			*Siderastrea siderea*	Partially Bleached
SR2	1	1	*Diploria labyrinthiformis*	Pale
MB1	5	4	*Agaricia agaricites*	Bleached
			*Dendrophyllia cylindricus*	Pale
			*Diploria strigosa*	Bleached
			*Diploria strigosa*	Partially Bleached
			*Siderastrea siderea*	Partially Bleached
MB2	5	5	*Agaricia agaricites*	Bleached
			*Agaricia humilis*	Bleached
			*Helioseris cucullata*	Partially Bleached
			*Isophyllia sinousa*	Pale
			*Manicina areolata*	Pale
MB3	4	4	*Agaricia agaricites*	Bleached
			*Diploria labyrnithiformis*	Pale
			*Manicina areolata*	Pale
			*Montastraea faveolata*	Pale
			*Mycetophyllia sp.*	Pale
PB	4	4	*Agaricia agaricites*	Bleached
			*Diploria labyrinthiformis*	Pale
			*Mycetophyllia sp.*	Pale
			*Porites porites*	Bleached
BRF	2	2	*Agaricia humilis*	Bleached
			*Agaricia agaricites*	Bleached
RDF	1	1	*Manicina areolata*	Pale

The severity of bleaching was classified according to the Atlantic and Gulf Rapid Reef Assessment (AGRRA) protocol as bleached, partially bleached and pale [25].

### Anthropogenic activity

No physical damage consistent with anchor usage or sand scour from shipping activity was noted at any of the sites assessed. Impact from anthropogenic activity was noted only at the MB1 site where several fish pots were encountered.

## Discussion

Coral species were documented at 16 out of 17 (or 94%) sites surveyed on Saba Bank and one site on Small Bank. As expected, coral species richness was higher on reef dominated areas as opposed to algal dominated ones. Algae are known to outcompete or replace coral for space via several mechanisms [11]. A total of 33 coral species were documented. Some specimens were not resolved past genus level (i.e. *Mycetophyllia* sp. and *Scolymia* sp.) and replicate vouchers in these two cases were not collected. Difficulty in resolving coral specimens to species level is not surprising given the phenotypic plasticity of corals.

According to the species accumulation curve (Fig. 2), more species of coral remain to be found. Most species are rare, so an asymptotic curve indicates that most of the common species were collected. Undocumented coral species are likely to be found through more exhaustive sampling effort in coral dominated reef habitats, at remote sites, and in deeper depths. Our study was designed to rapidly assess as many sites as possible utilizing only two roving scuba divers; therefore our sampling effort consisted of one dive per site. Some coral species may have been missed or overlooked. It is recommended that sampling effort be increased for future surveys to cover more area at each site. To explore deeper depths and extend bottom time, re-breathers and other technologies (e.g. ROV or submersibles) may prove useful.

Our study was spatially limited (18 sites), and it was far from comprehensive in coverage of the bank. Many more sites on Saba Bank remain to be explored and these may contain other coral species not documented here. Of particular note are the reefs on the south-western part of the Bank that have been sampled less relative to reefs in the northeastern part of the Bank [1], [2], [4]. The sampling bias toward the northeastern reefs is most likely a reflection of their proximity and accessibility for small boats traveling from Saba. More distant sites on further reaches of Saba Bank would require calm waters and/or larger vessels. To date, only Van der Land [2] visited the southwest portion of the Bank, operating from a hydrographic vessel. Our survey used small boats and was hampered by inclement weather.

The presence of healthy stands of *A. cervicornis* on five reefs sites was a particularly noteworthy aspect of the study. This species has been assessed as critically endangered on the IUCN red list [12], [13]. The Caribbean wide decline of this species due to disease has been well documented [14], [15]. It is recommended these stands and their surrounding habitat (reef sites CV2, MB1, MB3, SR1, and SR2) be given highest priority for full protection in the zoning use plan under development in consultation with all stakeholders.

The documentation of the invasive species *Tubastraea coccinea* was not surprising. Ship hulls are a potential vector [16], so maritime traffic and practice of anchoring on Saba Bank easily could have introduced this species. Saba Bank was noted in a study of the invasion of *T. coccinea* into the Gulf of Mexico [17]. Van der Land had reported *Tubastraea tenuilamellosa* (synonym of *T. coccinea* [18]) in the coastal waters of Saba Island in 1977 [2]. The introduction of invasive species via ballast water or ship hulls is an issue that needs to be addressed to enhance the protection and management of the marine resources of Saba Bank.

The lack of effects from bottom type and depth class on coral assemblages among sites suggests that either: 1) replication was insufficient, or 2) other factors not examined here are structuring the species composition. Differences in wave energy and exposure among sites may be the dominant factor influencing coral composition. A significant difference in species composition was evident among sites near and far from the platform edge, but sites did not group strictly according to class. The 25% dissimilarity of the SmB site to the others was expected given its isolation. Interestingly, SmB was more similar to the stony coral assemblages the reef sites on the bank then the NE reef site and the two plateau sites of SWC and RR located on the bank. The dissimilarity of RR and the NE sites to the other reef and plateau sites may be due to wave exposure given their position on the bank. The plateau site of SWC may be unique in its limited coral assemblage due to the dominance of this habitat by seaweed.

Coral richness is known to vary with depth and many environmental parameters (temperature and PAR) are correlated with depth [19]. Our depth range may have been too narrow to detect these differences. Moreover, replication was low in our deepest depth class. This supports the need to survey sites more comprehensively, especially deeper depths, and to explore more sites on the Bank.

More coral colonies exhibited bleaching on sites located in the northern to northeast side of Saba Bank compared to the other sites assessed. These sites were closest to Saba Island. No evidence of physical damage from anchors or other sources were noted at the sites surveyed. However, the potential for physical damage as well as other stressors due to maritime activity is high.

Although hurricanes and bleaching events are difficult to curtail, studies have shown that mitigation of other “manageable” stressors from anthropogenic activity may help to render coral reefs more resilient in the face of climate change [20]. Therefore sites with highest richness (GB, CV1, CV2, MB1, MB3, PB, SR1, and SR2), especially those with *A. cervicornis*, are recommended for full protection in the zone use plan for Saba Bank. The development and implementation of the plan should be done in full consultation with all stakeholders in order to be successful. Given the vast area of the bank and the spatial limitations of the present study, further exploration of the species and habitats on Saba Bank is warranted.

## Materials and Methods

The primary objective of this survey was to help document the biodiversity of select taxa on Saba Bank using rapid assessment techniques. In this study, only the richness of stony corals was reported. A severe bleaching event occurred prior to the survey [8] and the incidence of coral disease is increasing in the Caribbean [14], [15], so data on bleaching and disease were also collected. Evidence of physical damage to coral colonies consistent with anchoring practices or other anthropogenic activity was also examined.

### Site Selection

Sites to be assessed were chosen based on coarse resolution bathymetry, proximity to Saba Island, Van der Land's description [2], and qualitative information from reconnaissance dives on the Bank [1]. Given the large area of Saba Bank, the sites were intended to cover a broad range of localities with a focus on known or hypothesized reef areas. Reef areas were given top priority for sampling due to their likelihood of corals and their high susceptibility to physical damage from maritime activity [6], [7], [21].

Known reef areas occur on the east, southeast, southwest and southern edges of the bank. The deeper western side of Bank could potentially have patch reefs where “ridges” less than 30 m deep occur. Small Bank was included for purposes of exploration, because it lies within the Exclusive Economic Zone of The Netherlands, and reaches within 40 m of the surface. Lastly, areas located in the middle of Saba Bank (depths 20 m–30 m) were included to characterize the sandy habitat overlying rock pavement. Weather was another important factor during the survey period of the 4–15 January 2006. Wave heights were 3 m–4.5 m on several days.

### Field and Sampling Protocol

At each site, an underwater visual census by two roving scuba divers was carried out. Observed species were photo documented and recorded, along with dominant bottom type (pavement or reef). Observations on the incidence of bleaching or symptoms indicative of disease on coral colonies were also noted and photo documented. Any recently dead coral colonies were recorded and photo documented. Physical damage to coral colonies consistent with anchor damage or other evidence of anthropogenic activity was also recorded by site.

A collecting permit was issued to Saba Conservation Foundation through CITES and voucher specimens of each species observed were obtained for archival purposes. *A. cervicornis* was only photo-documented because it is a critically endangered IUCN red-listed species. Collection of specimens was kept to a minimum. Only one voucher specimen per species was collected in most cases. In some instances, more than one specimen was collected for comparison (i.e. examination of skeletal characters).

Specimens were collected by gently dislodging the colony at the base with hammer and chisel and immediately placing it into a sealable plastic bag filled with seawater. In the lab, specimens were soaked in a solution of freshwater mixed with a commercial bleach solution (12%NaOCl) to remove living tissue then the coral skeleton was dried and labeled. Identifications of coral specimens were made *in situ* and cross checked by comparing the photo image, skeleton and name to taxonomic references and field guides [22]–[25]. All specimens collected were packaged and shipped to Dr. S. Cairns and T. Coffer of the Smithsonian Museum of Natural History where taxonomic identification was verified or resolved. Specimens are curated at the Smithsonian (NMNH). Species documented during this survey were added to previous surveys on Saba Bank to generate a complete list of stony coral species.

To characterize the bleaching observed in coral colonies, the Atlantic and Gulf Rapid Reef Assessment Program methodology [26] was used. This entailed categorizing the severity of coral tissue discoloration as: pale for discoloration of coral tissue; partly bleached for patches of fully bleached or white tissue; and bleached for totally white tissue with no zooxanthallae visible. Observations and photos of coral diseases made during the survey were cross-checked with references and database images to confirm diagnosis [15], [27].

### Statistical Analysis

Survey completeness and total expected richness were assessed using an expected species accumulation curve with the non-parametric Chao1 and Jack 1 estimators [28] in EstimateS 8.0 software [29]. Sites were characterized by dominate substrate type (pavement or reef). Ten sites had substrates consisting of pavement, and they were classified by this bottom type. These included (GB), (MB1), (MB2), (MB3), (RR), (SWC), (SmB), (RVH), (RdF), and (BrF). The seven sites with reef as the dominant bottom type included (CV1), (CV2), (NE), (PB), (RB3), (SR1), and (SR2). Field of Greens (FoG) had sandy substratum. No corals were observed, so it was excluded from all subsequent analyses.

The survey sites were classified by depth as follows: deep ≥30 m, mid 25–29 m, and shallow <24 m). Depth measures were derived from SCUBA gauges. The deep class had three sites (GB, RR, and SmB). The mid-depth class included seven sites (CV1, MB2, NE, PB, RdF, RVH, and SR1). The shallow class had seven sites (BRF, CV2, MB1, MB3, RB3, SR2, and SWC).

Sites were also categorized as either reef or plateau, based on their distance from platform edge on Saba Bank, calculated using GPS coordinates in a Geographic Information System with high-resolution multi-beam data. The platform edge was defined as the seaward perimeter of the “reef crest” feature (Fig. 1). A two-zone scheme (reef or plateau) was used. Sites <500 m from the platform edge were called “reef”. Sites ≥500 m from the platform edge were called “plateau”. The six plateau sites included BrF, GB, RDF, RR, RVH, and SWC. The ten reef sites included CV1, CV2, MB1, MB2, MB3, NE, PB, RB3, SR1, and SR2. Small Bank (SmB), a seamount, was not categorized as either zone, it was treated as an independent site given its location off of Saba Bank.

To address the question of whether the species composition varied significantly with depth, distance, or habitat, a presence/absence data matrix was developed. Sites were the observations while coral species were the variables using values of 1 and 0 for presence and absence. Data were analyzed using the Plymouth Routines in Multivariate Ecological Research (PRIMER) version 6.1, software package [29]. A resemblance matrix based on the Bray-Curtis dissimilarity measure was calculated for all sites, and the resulting similarity/dissimilarity values were employed to generate multi-dimensional scaling (MDS) plots and group averaged hierarchical clustering dendrograms. Similarity contours were derived from the dendrograms and applied to the MDS plots in PRIMER 6.1.

The analysis of similarity (ANOSIM) [30] Global-R statistic [31] was employed to test the null hypothesis of no difference between groups of sites classified by substrate type (pavement or reef), by distance from the platform edge (reef is <500 m, plateau is >500 m) and by depth class (deep ≥36 m, mid 25–29 m, and shallow <24 m). ANOSIM *R* is a non-parametric permutation procedure that uses the underlying Bray-Curtis resemblance matrix to rank similarities among *a priori* designations. ANOSIM calculates a global R statistic from the fraction of the difference between average rank similarity *within* groups and average rank similarity *between* groups over a function of the number of samples. The resulting *R*-value ranges between 0 and 1, with high values indicating a large degree of discrimination among groups. Low R values can be significant if sample sizes are large.

The rationale of the ANOSIM permutation test is that there should be no effect on the average value of *R* when sites associated with samples are arbitrarily rearranged if there is no difference among sites. Sites are reshuffled in subsequent iterations to generate a null distribution of *R*. The number of permutations is a function of the number of sites and samples. The significance of *R* is calculated by referring the observed value of *R* to the spread of values generated by the random rearrangements. If the observed value is unlikely to come from the null distribution, the null hypothesis can be rejected [32].

The null hypothesis of no structure in the data between Caribbean localities was tested using the SIMPROF (similarity profile) routine on an underlying Bray-Curtis matrix. SIMPROF is a permutation-based ranking procedure designed to test *a posteriori* hypotheses of no difference. The underlying principle is that genuine data structure will be evidenced by more high and low similarity values than would be expected under the null hypothesis that all species are drawn from one assemblage. If the null is rejected, the species in the data pool are likely to come from different assemblages. MDS is generally preferred over cluster analysis because it preserves more of the original information in the dissimilarity matrix.

In order to determine whether the Saba Bank stony coral assemblage was typical of the Caribbean or unique, incidence data from Saba Bank were compared to published species lists from surveys that used similar techniques in the Bahamas [33] Colombia [34] and St. Lucia [35]. A second data matrix was assembled using localities as observations and coral species as variables, with n = 1 for each locality. A Bray-Curtis dissimilarity matrix was produced and a MDS plot was generated for graphic representation [30], but SIMPROF [10] was used to test the null hypothesis of no data structure among Saba Bank and the three Caribbean locations.
